# Wounding in the plant tissue: the defense of a dangerous passage

**DOI:** 10.3389/fpls.2014.00470

**Published:** 2014-09-16

**Authors:** Daniel V. Savatin, Giovanna Gramegna, Vanessa Modesti, Felice Cervone

**Affiliations:** Department of Biology and Biotechnology “Charles Darwin”, Sapienza–University of RomeRome, Italy

**Keywords:** wounding, oligogalacturonides, wound response, systemin, jasmonate

## Abstract

Plants are continuously exposed to agents such as herbivores and environmental mechanical stresses that cause wounding and open the way to the invasion by microbial pathogens. Wounding provides nutrients to pathogens and facilitates their entry into the tissue and subsequent infection. Plants have evolved constitutive and induced defense mechanisms to properly respond to wounding and prevent infection. The constitutive defenses are represented by physical barriers, i.e., the presence of cuticle or lignin, or by metabolites that act as toxins or deterrents for herbivores. Plants are also able to sense the injured tissue as an altered self and induce responses similar to those activated by pathogen infection. Endogenous molecules released from wounded tissue may act as Damage-Associated Molecular Patterns (DAMPs) that activate the plant innate immunity. Wound-induced responses are both rapid, such as the oxidative burst and the expression of defense-related genes, and late, such as the callose deposition, the accumulation of proteinase inhibitors and of hydrolytic enzymes (i.e., chitinases and gluganases). Typical examples of DAMPs involved in the response to wounding are the peptide systemin, and the oligogalacturonides, which are oligosaccharides released from the pectic component of the cell wall. Responses to wounding take place both at the site of damage (local response) and systemically (systemic response) and are mediated by hormones such as jasmonic acid, ethylene, salicylic acid, and abscisic acid.

## INTRODUCTION

The sessile condition exposes plants to any possible environmental stress. Injury, one of the most frequent stress conditions that plants must face, may cause both loss of nutrients and entry of microbes. Therefore, plants have evolved sophisticated mechanisms to promptly respond to wounding, rapidly heal the tissue and prevent microbe infections. Unlike animals, plants do not have mobile cells specialized for defense, but each plant cell has the capability to activate protective mechanisms upon injury sensing. The capacity of cells to activate defense responses upon “danger” sensing and recognition of non-self microbe-associated molecular patterns (MAMPs) and/or endogenous damage-associated molecular patterns (DAMPs) is characteristic of the plant innate immunity (Akira et al., 2006). Defense responses activated by wounding are similar and overlapping with those activated by MAMPs and DAMPs, indicating that both injury and pathogens are limited by plants in a similar manner. Most of our knowledge on wounding derives from studies in which plants are mechanically damaged. Mechanical injury activates defenses that are similar to those induced by herbivores and insects (Reymond et al., 2000; Arimura et al., 2005; Rehrig et al., 2014), although the damage caused by herbivores has peculiar characteristics and mechanical wounding is necessary but not sufficient to trigger the full response activated by insects (Maffei et al., 2007). Differences between mechanical wounding and attacks by herbivores and insects will be discussed in other reviews of this issue (Krautz et al., 2014).

Plants contrast wounding with both constitutive structures, such as epicuticular films and crystals of wax, and secretory conduits for latices or resins, that restrict the access of opportunistic microorganisms to the tissue, as well as wound-induced responses that, unlike the constitutive defenses, are energetically costly (Leon et al., 2001; Bonaventure and Baldwin, 2010) and thereby regulated and triggered only when required. The primary events of the response to wounding occur at the injured site (local response) while the undamaged tissues respond later (systemic response), upon perception of mobile signals that communicate the existence of a critical condition (Farmer and Ryan, 1992). Extracellular signals such as cell wall-derived oligogalacturonides (OGs) and peptides, like systemin, have been characterized as typical signals of wounding (Roberts, 1992). Genes involved in biosynthesis of jasmonic acid (JA) and ethylene (ET) as well as genes for general stress responses (oxidative stress, dehydration stress, heat-shock proteins, etc.) are rapidly induced (Reymond et al., 2000; Delessert et al., 2004). Later, events of protein turnover and transport processes involving aquaporins, lipid transfer proteins, ABC transporters, sugar, and peptide transporters occur. Finally, the modulation of primary metabolism (carbohydrate and lipid metabolism, nitrogen assimilation) and the expression of genes involved in the biosynthesis of secondary metabolites with repellent or anti-digestive activity [i.e., glucosinolates, cyanogenic glucosides, alkaloids, phenolics, and proteinase inhibitors (PI)] may occur. Transcriptional profiling analyses performed on 8.200 *Arabidopsis* genes revealed that approximately 8% of these genes are differential expressed after wounding and about 20% of the wounding-regulated genes encode proteins involved in signal transduction, such as members in the AP2, WRKY, and MYB families (Cheong et al., 2002). A large fraction of the wound-responsive genes are also responsive to pathogens, suggesting that signaling pathways activated by these stimuli are shared (see below). This clearly indicates that an important reprograming of gene expression occurs in plants to defend the damaged tissue, which represents an easy passage for pathogen invasion. Receptors and signal transduction elements usually involved in pathogen response as well as several putative disease resistance genes (*R* genes) are up-regulated by wounding (Cheong et al., 2002). This review is focused on what is currently known about the putative signals that are released upon wounding, on the mechanism of their perception and transduction and on plant defense responses activated upon injury sensing.

## SENSING THE WOUNDING THROUGH THE CELL WALL INTEGRITY

The cuticle, composed of cutin and cuticular waxes, covers the outermost epidermal cell wall in the aerial portions of plants (Riederer and Schreiber, 2001; Nawrath, 2006). The integrity of cuticle and cell wall (CWI) is affected by wounding and may be sensed by the plant cells. Studies on *Arabidopsis* plants expressing cutinase (Sieber et al., 2000) and on mutants impaired in cuticle biosynthesis have indicated that a more permeable cuticle allows the passage of diffusates with growth-inhibiting activity against phytopathogenic fungi (Bessire et al., 2007; Chassot et al., 2007). A breach in the cuticle caused by wounding also favors the diffusion of elicitors that, therefore, have an easier access to the cell surface, while an intact cuticle may prevent their passage from the surface. An early recognition of elicitors may lead to a prompt and efficient activation of the immune responses (L’Haridon et al., 2011; Benikhlef et al., 2013).

The cell wall is a dynamic structure that play a critical role in growth and development as well as in preventing wounding and pathogen attack (Bellincampi et al., 2014). The perception of an altered CWI is a key event during wounding (Nuhse, 2012; Wolf et al., 2012). Subtle modifications caused by physical perturbations such as light touch, soft mechanical stress, wind or contact with insects may be sensed at the level of the plasma membrane through stretch-activated mechano-sensitive channels that increase the intracellular Ca^2+^ concentration and trigger further signal transduction events (Nakagawa et al., 2007; Haswell et al., 2008; Benikhlef et al., 2013). Expression of the *Arabidopsis* calcium channels *MATING INDUCED DEATH 1* (MID1)-COMPLEMENTING ACTIVITY 1 (MCA1) and MCA2 in yeast complements the lethal effect of loss-of-function mutations in the *MID1* and *MID2* genes encoding stretch-activated calcium channels (Kanzaki et al., 1999; Ketela et al., 1999) and promote calcium influx upon mechanical stimulation (Nakagawa et al., 2007; Yamanaka et al., 2010). Putative cation channels belonging to the GLUTAMATE RECEPTOR-LIKE (GLR) family, which mediate calcium influxes in response to MAMPs (Kwaaitaal et al., 2011), are also required for the expression of several JA-inducible genes upon wounding in *Arabidopsis* (Mousavi et al., 2013).

Several *Arabidopsis* receptor like kinases (RLKs), among which those belonging to the *Catharanthus roseus* receptor-like kinase 1 family that includes THESEUS 1 (THE1), HERCULES 1, and FERONIA, have been proposed as possible sensors of CWI (Hematy et al., 2007; Guo et al., 2009). Loss of THE1 function attenuates the growth defects and ectopic lignification phenotype caused by a mutation in the *CELLULOSE SYNTHASE 6* (Hematy et al., 2007). Moreover, THE1 is involved in the accumulation of reactive oxygen species (ROS) and lignin deposition induced by isoxaben, an inhibitor of cellulose synthesis (Denness et al., 2011). This evidence clearly shows that defects in the cell wall caused by a disturbance of cellulose biosynthesis are sensed through THE1.

The monitoring of the status of pectin contributes to the sensing of CWI alterations (De Lorenzo et al., 2011). Plants carrying mutations that significantly alter pectin integrity, such as *quasimodo 2* or *tumorous shoot development 2*, exhibit constitutive induction of defense responses (Krupkova et al., 2007; Mouille et al., 2007). However, minor modifications in the methylation status, which occur in transgenic plants overexpressing the inhibitors of pectin methylesterases or in KO mutants of *PECTIN METHYLESTERASE 3*, do not influence the expression of defense genes (Lionetti et al., 2007, 2010, 2012; Raiola et al., 2011). Possible indicator of an altered pectin integrity is the presence of OGs, a well-known class of DAMPs that, similarly to MAMPs, act as danger signals for the activation of the immune responses (Boller and Felix, 2009; De Lorenzo et al., 2011). OGs are released from the plant cell walls upon partial degradation of homogalacturonan, the main component of pectin, by wound-induced hydrolytic enzymes or, during infections, by microbial hydrolytic enzymes. The size of OGs is critical for their elicitor activity, being OGs with a degree of polymerization (DP) between 10 and 15 most active while shorter oligomers are inactive. OGs induce in several plant species a wide range of defense responses, including production of ROS, nitric oxide, phytoalexins, glucanase, chitinase, and callose (Bellincampi et al., 2000; Galletti et al., 2008; Rasul et al., 2012; Ferrari et al., 2013). In tomato, OGs, probably generated by the action of a wound-inducible plant-derived polygalacturonase (PG; Bergey et al., 1999), induce the accumulation of PI (Ryan and Jagendorf, 1995). OGs may act only locally, because their oligoanionic nature confers them a limited mobility in the tissues (Baydoun and Fry, 1985). In *Arabidopsis*, both wounding and OG treatment induce a strong local resistance against the necrotrophic fungus *Botrytis cinerea* that is independent of salicylic acid (SA)- and JA-mediated signaling (Chassot et al., 2007; Ferrari et al., 2007). OGs also antagonize auxin responses (Branca et al., 1988; Bellincampi et al., 1996; Ferrari et al., 2008; Savatin et al., 2011), but the auxin-OG antagonism is uncoupled from their activity as defense elicitors. Indeed, the *Arabidopsis* mitogen-activated protein (MAP) kinase kinase kinases ANPs have been identified as elements in the OG-mediated induction of defenses, but do not play a major role in the inhibition of the auxin-induced gene expression (Savatin et al., 2014).

OG sensing in *Arabidopsis* may involve wall-associated kinases (WAKs; Brutus et al., 2010; Kohorn and Kohorn, 2012). WAKs are RLKs consisting of an extracellular domain, containing epidermal growth factor repeats, a transmembrane domain and a cytoplasmic Ser/Thr kinase domain. The extracellular domain of WAK1 and WAK2 also contains an N-terminal portion that binds pectin *in vitro* (Decreux and Messiaen, 2005; Kohorn et al., 2009). OGs with a DP > 9 bind reversibly WAK1 and the binding increases when OGs are present as dimers in a calcium-mediated “egg box” conformation (Decreux and Messiaen, 2005; Cabrera et al., 2008). Five *WAK* genes are clustered on chromosome 1 and additional 21 *WAK-like* genes (*WAKL*) are present in *Arabidopsis* (Verica et al., 2003). *WAK1*, *WAK2*, *WAKL5*, and *WAKL7* are induced by wounding (Wagner and Kohorn, 2001; Verica et al., 2003). In rice, *OsWAK1* is also induced by mechanical wounding as well as by SA and methyl-JA (MeJA) but not by abscisic acid (ABA; Li et al., 2008). A role of WAK2 in the wound response was suggested by the analysis of gene expression in plants overexpressing WAK2 fused to a TAP epitope (Kohorn et al., 2012).

A lectin receptor kinase-I.9 (DORN1), which plays a role in the perception of extracellular ATP, is also involved in the wound responses. Indeed, ectopic expression of DORN1 enhances expression of genes co-regulated by wounding and ATP (Choi et al., 2014). A maize wound-induced gene encoding a leucine-rich RLK (WPK1) is involved in JA- and phytochrome-mediated signaling (He et al., 2005). In tobacco, a leucine-rich repeat RLK (WRK) is involved in the JA-dependent wound signaling and acts upstream of the SA- and wound-induced protein kinases SIPK and WIPK, respectively (Seo et al., 1995; Zhang and Klessig, 1998a,b; Takabatake et al., 2006). WRK expression increases 15 min after wounding (Ito et al., 2002). WRK orthologs are present in dicots (*Arabidopsis* and tomato) but not in monocots (rice and wheat; Takabatake et al., 2006).

## WOUND-ASSOCIATED DAMPs

Peptides that function as DAMPs have been isolated in wounded tissues. Systemin, a 18-aminoacid peptide, was identified in tomato after wounding or insect attack as a cleavage product released into the apoplast from prosystemin, i.e., a larger cytoplasmic precursor protein that accumulates in the cytosol of phloem parenchyma cells (Jacinto et al., 1997; Narvaez-Vasquez and Ryan, 2004; Schilmiller and Howe, 2005). Sensing of systemin activates the biosynthesis of JA, which, in turn, activates defenses responses in neighboring cells (Orozco-Cardenas et al., 1993). The systemin receptor was identified as the tomato homolog of the brassinosteroid receptor BRI1, SR160 (Scheer and Ryan, 1999), but more recent findings argued against this evidence (Hind et al., 2010). Hydroxyproline-rich systemins (HypSys) that trigger plant immunity during herbivore or pathogen attack (Heiling et al., 2010; Bhattacharya et al., 2013) have been identified in Solanaceae (Pearce et al., 2001, 2007, 2009; Pearce and Ryan, 2003; Bhattacharya et al., 2013) and in sweet potato (Chen et al., 2008). HypSys peptides, as systemin, are processed from precursor proteins which are induced by wounding (Narvaez-Vasquez et al., 2005).

A peptide, Pep1, was identified in *Arabidopsis* for its capability of inducing alkalinization in suspension-cultured cells. Pep1 is a 23-amino acid peptide released from the C-terminus of a 92 amino acid precursor protein, PROPEP1, which is induced by wounding, MeJA and ET. *PROPEP1* belongs to a gene family of eight members. The family members *PROPEP2* and *PROPEP3*, and, to a lesser extent, *PROPEP1* are strongly induced by microbial pathogens such as *B. cinerea*, *Phytophthora infestans*, and *Pseudomonas syringae* as well as by various MAMPs and DAMPs elicitors, including NPP1, HrpZ, flg22, and OGs (Craigon et al., 2004; Toufighi et al., 2005; Denoux et al., 2008). PROPEPs are localized in the cytosol and the tonoplast and may function in the amplification/modulation of elicitor-triggered responses rather than being signals responsible for the initiation of the defense responses (Huffaker et al., 2006; Bartels et al., 2013). Homologues of AtPeps have been identified in maize. ZmPep1 regulates disease responses whereas ZmPep3 triggers the biosynthesis of JA and ET and induces the production of anti-herbivore volatiles (Huffaker et al., 2011, 2013). AtPeps are perceived by two RLKs (PEPR1 and PEPR2), which share structural and functional similarity to the MAMP receptors FLS2 and EFR (Yamaguchi et al., 2006; Krol et al., 2010). PEPR1 and PEPR2 are induced by wounding and MeJA but not by SA and 1-aminocyclopropane-1-carboxylic acid (ACC) synthase (Yamaguchi et al., 2010). They are also differentially induced by DAMPs (AtPeps and OGs) and MAMP (flg22 and elf18; Zipfel et al., 2004, 2006; Denoux et al., 2008; Yamaguchi et al., 2010).

Cutin monomers, that are formed as a breakdown of the cuticle, have been proposed as signal molecules for the induction of disease resistance in cereals, i.e., barley and rice (Schweizer et al., 1994). Fungal pathogens such as *Erysiphe graminis* and *Magnaporthe grisea* are able to produce and secrete cutinases that facilitate the formation of cutin monomers in the infection site. Pretreated barley leaves with cutin monomers display acquired protection against *E. graminis* (Schweizer et al., 1996b) and evidences that free cutin monomers can be recognized by plant cells as endogenous stress-related signals were obtained in cultured potato cells (Schweizer et al., 1996a).

## SIGNAL TRANSDUCTION UPON WOUNDING

Many events triggered by wounding have been uncovered and are discussed here.

### ELECTRIC SIGNALS

The involvement of electrical signals in the local and systemic alert in plants was postulated in 1992, when it was found that mechanical wounding in tomato cotyledons causes the transmission of a potential action to the first unwounded leaf concomitantly with the induction of PI proteins at the site of injury (Wildon et al., 1992). More recently, it has been shown that mechanical wounding at the tips of *Arabidopsis* leaves generates, within a few seconds, wound-activated surface potential (WASP) changes that are consequent to a plasma membrane depolarization. The WASP signal first moves from tips toward the center of the rosette leaves and then to a restricted and selected number of distal leaves. For example, wounding at the tip of leaf no. 8 causes WASP changes of the same amplitude and duration in leaves no. 5, 11, 13, and 16 but not in other leaves. Both JA and JA-responsive gene expression increases with a total of 313 genes up-regulated both locally and systemically. GLR proteins, putative cation channels, are required for WASP propagation leading to defense gene expression (Mousavi et al., 2013). Similar WASP effects on JA levels and defense gene expression have been reported in tomato plants upon wounding (Herde et al., 1996). Electric signals that propagate over distances of 100 cm from the wounded site are generated in avocado trees (Oyarce and Gurovich, 2011).

### ION FLUXES

One of the earliest responses (0.5–2 min) activated by the elicitors of plant defenses is the membrane depolarization following the influx of H^+^ and Ca^2+^ and a concomitant eﬄux of K^+^ and nitrate across the membrane (Nurnberger et al., 2004; Mithofer et al., 2005). Injury-induced ion fluxes occur in both dicots such as *Vicia faba* and monocots such as *Hordeum vulgare* (Zimmermann et al., 2009). Calcium spikes are critical for downstream signaling, since the physiological concentration of cytosolic calcium very rapidly increases after “danger” sensing (Lecourieux et al., 2006; Kudla et al., 2010; Reddy et al., 2011). In plants, as in animals, calcium is a well-known second messenger. Plants discriminate among the various stimuli by generating “calcium signatures” that are characteristic in terms of sub-cellular localization, amplitude, duration and frequency (Sanders et al., 2002). Intracellular peaks of calcium are detected in both epidermis and vascular cells proximal to the injury within 6 s (Beneloujaephajri et al., 2013). Signatures are decoded by three major types of sensor proteins: calmodulins (CAMs) and CAM-like proteins, calcineurin B-like proteins (CBL) and calcium-dependent protein kinases (CDPKs), a class of calcium sensors bearing both protein kinase and CAM-like domains in a single polypeptide (Luan et al., 2002; Harper and Harmon, 2005; Luan, 2009). Different studies highlight the role of calcium sensors in plant immunity. For example, the *Arabidopsis* CAM binding protein (CBP) 60 g contributes to flg22-induced accumulation of SA and is involved in resistance against *P. syringae* (Wang et al., 2009); the rice CBL-interacting protein kinases (CIPKs) 14 and 15 are involved in various MAMP-induced immune responses (Kurusu et al., 2010); the potato and tobacco CDPKs participate in the activation of the oxidative burst (Ludwig et al., 2005; Kobayashi et al., 2007, 2012). In *Arabidopsis*, CALCIUM-DEPENDENT PROTEIN KINASE 3 (CPK3) and CPK13 are required for defense gene induction upon feeding by the generalist herbivore *Spodoptera littoralis* (Kanchiswamy et al., 2010). CPK3 is also activated by flg22 in *Arabidopsis* protoplasts suggesting that it is involved in MAMP signaling as well (Boudsocq et al., 2010). In tomato, LeCDPK2 contributes to wound-triggered ET production by phosphorylating and activating the ET biosynthesis enzyme ACC SYNTHASE 2 (Kamiyoshihara et al., 2010). In *Nicotiana attenuata*, CDPK4 and CDPK5 are negative regulators of JA synthesis; plants with silenced expression of these two CDPKs are more resistant to larvae of *Manduca sexta* and exhibit enhanced responses to mechanical wounding (Yang et al., 2012). MeJA, touching and mechanical wounding enhance a calcium-activated CDPK activity that induces systemic wound responses also in maize (Szczegielniak et al., 2012).

### REACTIVE OXYGEN SPECIES

The production of ROS is a highly conserved process among aerobic organisms and is involved in defense and development processes of plants. ROS are emerging as signal molecules in plant immunity activation in response to both pathogens and wounding (Mittler et al., 2011; Suzuki and Mittler, 2012). In tomato, hydrogen peroxide is detected within 1 h after wounding and increases at 4–6 h both locally and in the upper unwounded leaves. OGs generated by a plant PG probably act as mediators of this process. A tomato mutant unable to properly respond to wounding neither induce PG nor generates hydrogen peroxide and is more susceptible to larvae of *Manduca sexta* (Orozco-Cardenas and Ryan, 1999). Given its toxicity, hydrogen peroxide must be tightly regulated to work as a signal molecule, and this is achieved through a complex mechanism involving calcium, protein phosphorylation, and production of ROS-scavenging enzymes that determine its steady-state levels in the cell. Wound-induced apoplastic hydrogen peroxide is produced by transmembrane NADPH oxidases (RBOHs) and by peroxidases, which also have a role in detoxification of other ROS (Minibayeva et al., 2014). The C-terminal region of plant RBOHs contains cytosolic FAD- and NADPH-binding domains and six conserved transmembrane domains while the cytosolic N-terminal region contains two EF-hand motifs which bind calcium (Kobayashi et al., 2007; Oda et al., 2010; Proels et al., 2010; Kimura et al., 2012; Drerup et al., 2013). Indeed, the wound-induced oxidative burst is dependent on calcium spikes and occurs also in the absence of the stimulus through artificially increasing the calcium levels in the cells (Monshausen et al., 2007; Takeda et al., 2008; Kimura et al., 2012). On the other hand, wound-related production of hydrogen peroxide is abolished by pretreatments with the calcium channel blocker verapamil or calcium chelators EGTA and oxalate (Beneloujaephajri et al., 2013). A calcium-dependent protein kinase CPK5 phosphorylates RBOHD and, probably, represents the link between calcium accumulation and ROS production. CPK5 phosphorylates *in vitro* and *in vivo* the N-terminal serine residues S39, S148, S163, and S347 of AtRBOHD (Dubiella et al., 2013) while, in a contradicting report, ROS production triggered by pathogen infection is reduced in *cpk1 cpk2* double mutant plants (Gao et al., 2013). RBOHD forms complexes with EFR and FLS2 as well as with the plasma membrane-associated kinase BOTRYTIS-INDUCED KINASE 1 (BIK1), which is also required for the protection conferred by wounding against pathogens (Laluk et al., 2011). BIK1 directly interacts with and phosphorylates different residues of RBOHD in response to elicitors (Kadota et al., 2014). In addition, RBOHF activity is regulated both through direct binding of Ca^2+^ to EF-hands and through calcium-dependent phosphorylation by CBL1/9-CIPK26 complexes (Drerup et al., 2013).

### MITOGEN-ACTIVATED PROTEIN KINASES (MAPKs)

Mitogen-activated protein kinase cascades amplify several abiotic and biotic stimuli leading to appropriate physiological responses (Rodriguez et al., 2010). They consist of a core module of three kinases that perform sequential phosphorylation reactions: a MAP kinase kinase kinase (MAP3K) activates a MAP kinase kinase (MAP2K), which activates a MAPK. Involvement of MAPKs in wounding has been widely described in various plant species (Nakagami et al., 2005). In *Arabidopsis*, wounding activates MEKK1, MPK3, MPK19 and, consequently, MEKK1 phosphorylates MKK1 (Hadiarto et al., 2006). Wounding also activates MPK4 and MPK6 and plants overexpressing the PP2C-type phosphatase AP2C1, which dephosphorylates and inactivates MPK4 and MPK6, do not respond to wounding (Ichimura et al., 2000). On the contrary, *ap2c1* mutants display enhanced responses to wounding and are more resistant to phytophagous mites (*Tetranychus urticae*; Schweighofer et al., 2007). On the other hand, MPK8, which is activated through direct binding of CAMs in a Ca^2+^-dependent manner and through a MKK3-mediated phosphorylation, negatively regulate the expression of RBOHD and ROS homeostasis triggered by wounding (Nemoto et al., 2011). In tobacco, the SA-induced protein kinase kinase SIPKK and MPK4, which are orthologs of *Arabidopsis* MKK1/MKK2 and MPK4, respectively, are required for wound-induced expression of JA-responsive genes, being MPK4 activated by SIPKK (Gomi et al., 2005). Moreover, WIPK and SIPK, which are orthologs of the *Arabidopsis* MPK3 and MPK6, respectively, are also involved in wounding signaling (Seo et al., 2007).

### HORMONES

Wounding induces *de novo* synthesis of JA, ABA, and ET, which are known to activate a network of interconnected pathways that coordinate host defense responses (Peña-Cortés et al., 1995; Bergey et al., 1996; Bouquin et al., 1997). JA accumulates in wounded plants and activates expression of various defense genes such as those encoding PI, thionin, and enzymes involved in secondary metabolism (Creelman and Mullet, 1997). Jasmonates, including the active form jasmonoyl-isoleucine (JA-Ile), derive from plastidial fatty acids through at least 10 intermediates and the involvement of three cellular compartments (Staswick and Tiryaki, 2004; Browse, 2009; Fonseca et al., 2009; Schaller and Stintzi, 2009). In *Arabidopsis*, wounding at leaf no. 8 promptly (90 s) induces an increase of JA amount in leaf no. 13, which shares a connected vasculature with leaf no. 8 (Dengler and Kang, 2001). Plant 13-lipoxygenases (13-LOXs) catalyze the first event in JA synthesis, i.e., the dioxygenation of fatty acids (Andreou and Feussner, 2009). In *Arabidopsis*, LOX2 is required for the JA synthesis proximal to the wound (Glauser et al., 2009) while LOX6 is required for JA and JA-Ile accumulation in the wounded as well as in the distal unwounded leaves. The conversion of JA to JA-Ile takes 50 s in the wounded leaf and about 100 s in the distal connected leaf no. 13 (Chauvin et al., 2013). JA and JA-Ile accumulation in response to wounding have been demonstrated to be dependent on WASPs (see above, Mousavi et al., 2013). Another important step in JA synthesis is the accumulation of the JA precursor 12-oxo-phytodienoic acid (OPDA) catalyzed by allene oxidase (AOS), which is induced by tissue injury (Leon et al., 2001). In potato, two putative AOS genes, StAOS1 and StAOS2, are differentially induced upon wounding and are required for OPDA and JA accumulation both in wounded and in unchallenged tissues (Taurino et al., 2014). A possible link between JA signaling and CWI alterations is suggested by the analysis of the *Arabidopsis cev1* and *cob* mutants, which have defects in cellulose synthesis and deposition and produce higher amount of JAs (Ellis et al., 2002; Ko et al., 2006). On the other hand, plants with a reduced expression of StAOS1 and StAOS2, and, consequently, a lower amount of OPDA, display reduced PME activity, increased methyl esterification level of pectins and an increased susceptibility to an hypovirulent strain of *Dickeya dadantii* (Taurino et al., 2014). In tomato, JA is preferentially generated in vascular bundles and accumulates in the midrib of leaves (Stenzel et al., 2003). AOS and lipoxygenases are located in the companion-cell–sieve-element complex of the vascular bundle (Hause et al., 2003). Since systemin accumulates in phloem parenchyma cells (Narvaez-Vasquez and Ryan, 2004) and activates the octadecanoid pathway for JA biosynthesis, it may be hypothesized that perception of systemin on the surface of companion cells initiates the synthesis of JA that is rapidly transported along the phloem (Schilmiller and Howe, 2005).

Abscisic acid is a stress hormone that mediates plant responses to drought and salinity (Finkelstein, 2013) as well as the expression of wound-induced *PROTEINASE INHIBITOR II* (*PIN2*) gene in tomato and potato (Peña-Cortés et al., 1995). Moreover, ABA positively regulates programmed death in *Arabidopsis* leaf cells surrounding the wounding site to confine injury and/or pathogen infections (Bostock and Stermer, 1989). Spreading of programmed cell death from wounded sites is repressed by the transcription factor MYB108 or BOTRYTIS SENSITIVE1 (BOS1; Mengiste et al., 2003), and plants lacking this element exhibit mis-regulated cell death after wounding (Cui et al., 2013).

Ethylene production upon wounding has been documented (O’Donnell et al., 1996; Bouquin et al., 1997; Liu et al., 1997). Among the early wound-induced genes there are several ACC synthase genes and many of ET response transcription factors, i.e., EREBPs (Cheong et al., 2002). In tomato, ET and wound signaling, mediated by systemin and JA, have been reported to independently act on resistance against *B. cinerea* (Diaz et al., 2002). ET and JA, besides mediating inducible defenses in response to wounding, have been also proposed to function in the trade-off between growth and defense and the associated changes in resource allocations (Onkokesung et al., 2010).

### LATE RESPONSES

Early intra- and inter-cellular events activated around the wounded site are required for late responses such as deposition of callose, suberin, lignin, and synthesis of various phenolics that may function both as a physical barrier and as antimicrobial substances. An *Arabidopsis* callose synthase, PMR4, is required for wound-induced callose formation (Jacobs et al., 2003). Callose is a (1→3)-β-D-glucan synthetized in all types of plant tissues in response to wounding (Chen and Kim, 2009). Its accumulation is dependent on the oxidative burst (Daudi et al., 2012; O’Brien et al., 2012) and occurs at the level of the cell wall either at wounded penetration sites or during attempted infections of fungi (Bellincampi et al., 2014). Callose may also prevent the spread of viruses through plasmodesmata (Benitez-Alfonso et al., 2011). Activity of both copper amine oxidases (CuAO) and flavin-containing amine oxidases (PAO), hydrogen peroxide-producing enzymes responsible for the oxidative de-amination of polyamines, appears to be important in wound healing in tobacco plants (Tisi et al., 2008). CuAO mediates also the enhanced accumulation of cell wall phenolics, observed on wound surface in tobacco plants over-expressing a fungal endopolygalacturonase, which show constitutively activated defenses. This observation suggests an important role of polyamine catabolism-derived hydrogen peroxide in the response activated by a compromised pectin integrity (Cona et al., 2014). Reconstruction of damaged tissues often takes place upon wounding and involves vascular and/or other cells that may divide and differentiate to reunite the existing tissues. Moreover, active biosynthesis and accumulation of pectic substances has been described in the cell wall of the reunion region in the cortex in cucumber and tomato hypocotyls (Asahina et al., 2002). The transcription factors RAP2.6L and ANAC071 are induced by ET and JA, differentially expressed around the injury site and are essential for tissue reunion of *Arabidopsis* wounded flowering stems (Asahina et al., 2011).

## CONCLUSION

**FIGURE 1:**
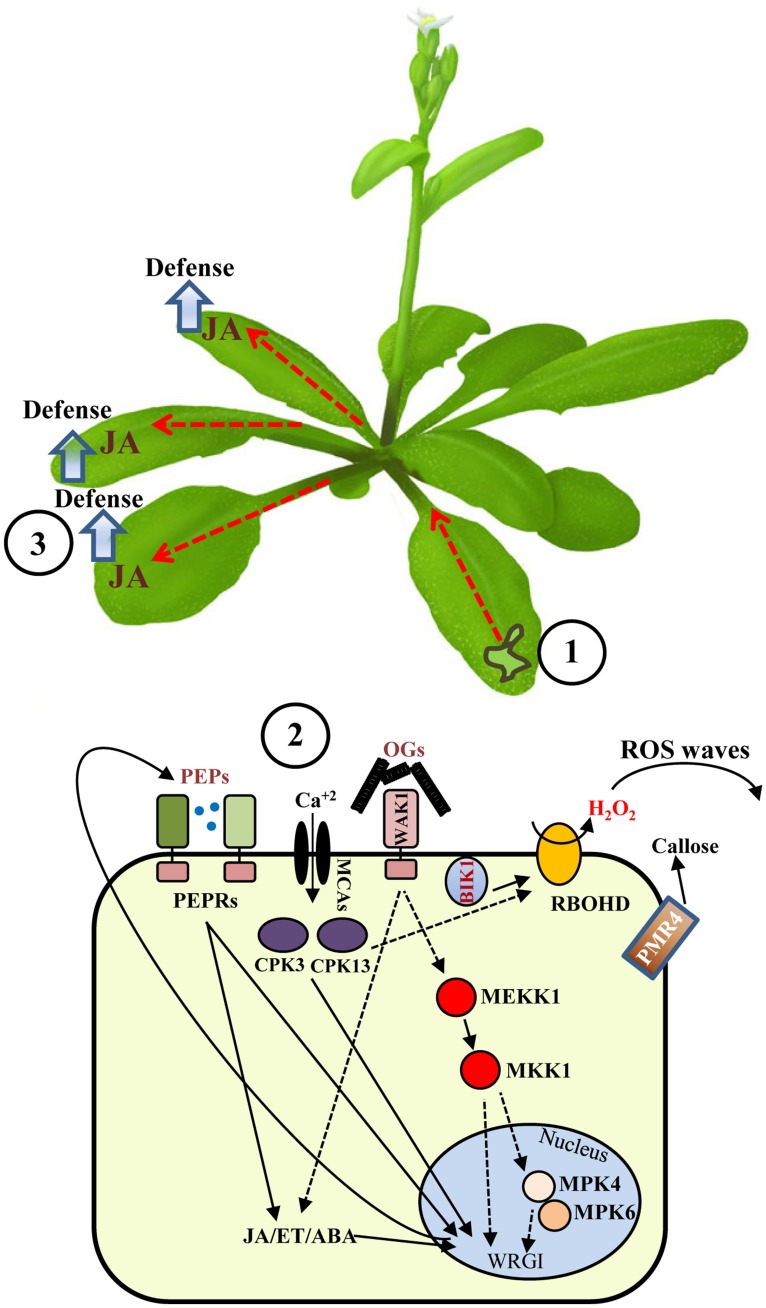
**Local and systemic responses induced by wounding in *Arabidopsis*.** Wounding of *Arabidopsis* leaves (1) is sensed through mechano-sensitive elements or by recognition of damage-associate molecular patterns, such as OGs or PEPs, which are perceived at the plasma membrane level. (2) Elements involved in wound signaling include calcium channels, MAPK cascades, CDPKs, and other kinases. Cell-to-cell communication is achieved by H_2_O_2_ waves produced by the trans-membrane NADPH oxidase RBOHD. Alert messages are generated and systemically propagated to undamage tissues through WASPs (red dashed lines) and other signals, such as JA. (3) Dashed lines indicate still partially uncharacterized roles of MAPKs or hypothetical cascades. WRGI: wound-regulated gene induction.

Pathogens often utilize wounded tissues for their entry into the plant. Wounding is rapidly perceived through an efficient surveillance mechanism of tissue integrity followed by cell-to-cell communication and long-distance signaling. Every cell is able to rapidly produce and propagate different alert messages, such as WASPs and ROS waves (**Figure 1**), which rapidly prime the rest of the plant to set up defenses against the potential danger. Propagation occurs over long distances, between different parts of the same plant and even between different individuals through volatile molecules production (Komarova et al., 2014). In the recent years several elements involved in sensing and signaling of wounding have been identified showing that the defense-related responses activated by wounding are comparable and almost overlapping with those activated after elicitor sensing. Thus, injury triggers a similar level of alert as a pathogen does, indicating that a breach in the physical barriers of the plant needs to be efficiently defended.

## AUTHOR CONTRIBUTIONS

Daniel V. Savatin and Giovanna Gramegna contributed equally to the manuscript. Daniel V. Savatin, Giovanna Gramegna, and Vanessa Modesti wrote the initial draft of the manuscript. Daniel V. Savatin designed and drew (**Figure 1**). All authors discussed the content of the manuscript.

## Conflict of Interest Statement

The authors declare that the research was conducted in the absence of any commercial or financial relationships that could be construed as a potential conflict of interest.
